# Exploring the Mechanisms of a Patient-Centred Assessment with a Solution Focused Approach (DIALOG+) in the Community Treatment of Patients with Psychosis: A Process Evaluation within a Cluster-Randomised Controlled Trial

**DOI:** 10.1371/journal.pone.0148415

**Published:** 2016-02-09

**Authors:** Serif Omer, Eoin Golden, Stefan Priebe

**Affiliations:** Unit for Social and Community Psychiatry, WHO Collaborating Centre for Mental Health Services Development, Queen Mary University of London, London, United Kingdom; University of California, Los Angeles, UNITED STATES

## Abstract

**Background:**

DIALOG+ is a new intervention to make routine community mental health meetings therapeutically effective. It involves a structured assessment of patient concerns and a solution-focused approach to address them. In a randomised controlled trial, DIALOG+ was associated with better subjective quality of life and other outcomes in patients with psychosis, but it was not clear how this was achieved. This study explored the possible mechanisms.

**Methods:**

This was a mixed-methods process evaluation within a cluster-randomised controlled trial. Focus groups and interviews were conducted with patients and clinicians who experienced DIALOG+ and were analysed using thematic analysis. The content of DIALOG+ sessions was recorded and analysed according to (i) the type of actions agreed during sessions and (ii) the domains discussed. The subjective quality of life measure was analysed with mixed-effects models to explore whether the effect of DIALOG+ was limited to life domains that had been addressed in sessions or consistent across all domains.

**Results:**

Four qualitative themes emerged regarding the mechanisms of DIALOG+: (1) a comprehensive structure; (2) self-reflection; (3) therapeutic self-expression; and (4) empowerment. Patients took responsibility for the majority of actions agreed during sessions (65%). The treatment effect on subjective quality of life was largest for living situation (accommodation and people that the patient lives with) and mental health. Two of these domains were among the three most commonly discussed in DIALOG+ sessions (accommodation, mental health, and physical health).

**Conclusion:**

DIALOG+ initiates positive, domain-specific change in the areas that are addressed in sessions. It provides a comprehensive and solution-focused structure to routine meetings, encourages self-reflection and expression, and empowers patients. Future research should strengthen and monitor these factors.

**Trial Registration:**

ISRCTN Registry ISRCTN34757603.

## Introduction

A central component of community mental health care is the routine meetings between a patient and their dedicated clinician (often called a key worker or care coordinator) to assess ongoing needs and coordinate care. Until recently, there was no evidence-based model on how to make these meetings therapeutically effective [1, 2]. While a number of previous interventions attempted to improve routine meetings by feeding back regular outcome data to patients and clinicians, they had little impact on patient outcomes [3–5]. This may be because they merely provided information without successfully influencing the behaviour of clinicians and patients during the meetings.

To address this, DIALOG+ was developed as an evidence-based model to make routine community meetings therapeutically effective. It is a computer-mediated intervention in which patients are invited by clinicians to rate their satisfaction with eight life domains (e.g. mental health, physical health, job situation) and three treatment aspects (e.g. meetings with professionals, medication) on a tablet computer. Following this, clinicians use a 4-step approach based on Solution Focused Therapy to discuss concerns arising from the ratings and identify actions for change. A recent cluster-randomised controlled trial compared DIALOG+ to an active control in patients with psychosis [6]. A cluster design was used to avoid contamination from the practice of clinicians. Even with just three sessions over 12 months on average, DIALOG+ was associated with better subjective quality of life (primary outcome, SQOL) after three, six and 12 months. The effect was equivalent to an improved rating on at least three out of 12 SQOL domains (Cohen’s d = 0.29–0.34) and comparable to much more intensive treatments, including Cognitive Behavioural Therapy [7, 8]. It was also associated with fewer unmet needs, lower symptom levels, better objective social outcomes, and lower treatment costs.

Possible mechanisms have been suggested as to why this intervention was so effective in improving patients’ SQOL [6]. In contrast to previous interventions, DIALOG+ may directly structure clinician and patient behaviour within the meetings. This includes the initial assessment of the patient’s concerns followed by the 4-step approach to facilitate solutions in the identified areas. Through this process it may have a domain-specific effect in identifying a problem and initiating real change in the patient’s life. Additionally, DIALOG+ adopts a solution-focused approach which focuses on the patient’s own resources and strengths [9]. This is important given that existing research has found that the majority of actions agreed during community meetings focus on what clinicians can do to help (71% of actions), with the patient taking responsibility for only a minority (8% of actions) [10]. DIALOG+ may encourage the patient to draw on their own resources and take more responsibility for their treatment.

In line with the Medical Research Council framework for developing and evaluating complex interventions [11, 12], the present study aimed to conduct a mixed-methods process evaluation to understand the effect of DIALOG+ on SQOL in the aforementioned trial. Process evaluations may focus on implementation, mechanisms, or context [12]. The present study focused only on exploring the possible mechanisms through which DIALOG+ was effective in order to inform the theory, practice and further development of DIALOG+. Informed by the anticipated mechanisms above, the specific objectives were:

1. To explore the views of patients and clinicians that experienced DIALOG+ to identify possible mechanisms.
2. To explore the nature of the action items agreed in DIALOG+ sessions, including the type of solutions and the person responsible for them.
3. To explore whether the treatment effect on SQOL was domain-specific to the topics addressed in DIALOG+ sessions, or a consistent effect across all domains.

## Methods

### Design

This is a mixed-methods process evaluation nested within a cluster-randomised controlled trial that tested the effectiveness of DIALOG+ in the community treatment of patients with psychosis [6, 13]. Guidelines for process evaluations were followed [12, 14]. A mixed methods approach was taken to enhance the interpretation of the findings in that separate analyses could be integrated, complement one another, and counter the others’ weaknesses [15]. Conducted in the following sequence, it included: (1) a thematic analysis of the views of patients and clinicians; (2) a content analysis of DIALOG+ sessions; and (3) a quantitative analysis of individual domains on the SQOL outcome. In line with the protocol, data collection was carried out in parallel to the main trial and prior to awareness of the outcomes. However, these additional analyses were carried out post-hoc to retrospectively explore the mechanisms. Each analysis was conducted and reported independently in the above sequence and researchers were aware of the findings of prior analyses. The findings were later integrated and interpreted by the research team (reported in the discussion) to generate hypotheses.

Detailed methodology of the trial design is reported in the protocol [13] and primary paper [6]. It was an exploratory, pragmatic, parallel group, cluster-randomised controlled trial. Clinicians were allocated to either DIALOG+ or an active control once all participating patients under their care had been recruited and baseline assessments completed. Clinicians were randomised with an allocation ratio of 1:1 by an independent statistician using computer generated randomisation lists, with allocation concealed from outcome assessors. The pre-specified target sample of 36 clinicians was increased to 49 as a lower number of patients were being recruited than expected. For organisational reasons the trial was registered after enrolment of the first participant (Controlled Trials number: ISRCTN34757603). However, this does not influence the integrity of the trial as registration took place before the analysis plan was written (and signed off by the Principle Investigator and trial statistician after approval by the independent Data Monitoring and Ethics Committee) and before any unblinding or analysis. The authors confirm that all their ongoing trials are registered.

### Participants and setting

The trial was conducted in Community Mental Health Teams across East London, where each patient has a dedicated clinician whom they meet about once a month to coordinate their care. Teams and clinicians were identified by the management organisation (East London NHS Foundation Trust) to reflect the pragmatic nature of the trial. Clinicians were eligible for the trial if they had a professional qualification, more than six months’ experience of working in community mental health care, and no plans to leave their post within the study period.

Eligible patients, under the care of participating clinicians, were approached in a random pre-defined order until the target cluster size was reached. Patients were included if they met the following criteria: age of 18–65 years; treatment in the community team for at least one month; no planned discharge for the next six months; a clinical diagnosis of schizophrenia or a related disorder (ICD-10 F20-29); and capacity to give informed consent. They were excluded if they had a mean score of 5 or higher on the Manchester Short Assessment of Quality of Life (MANSA) [16], reflecting an average rating of at least ‘mostly satisfied’ with all life domains, and if they had insufficient command of English. Recruitment took place between October 2012 and September 2013, with follow-up data collection between February 2013 and October 2014. Detailed recruitment procedures are reported elsewhere [6, 13]. Written informed consent was obtained from all clinicians and patients. The study received a favourable opinion from the National Research Ethics Service (NRES) London, Stanmore (12/LO/1145).

### Interventions

#### Experimental condition

In the experimental group, clinicians and patients were instructed to use DIALOG+ once per month over a six-month period and flexibly thereafter at their discretion. DIALOG+ is delivered using a tablet computer throughout to display each stage visually and record the outcome of the session. It includes an initial assessment of the patient’s satisfaction with eight life domains (e.g. mental health, physical health, job situation) and three treatment domains (medication, practical help, meetings with professionals) on a Likert scale. This is followed by a review of the ratings, positive feedback on high-scoring domains, and selection of the domains to discuss further in the meeting. Finally, a 4-step solution-focused approach is used to discuss the chosen domain and identify actions. The steps include (1) understanding the negative and positive aspects of the situation, (2) looking forward towards solution-focused goals, (3) exploring what the patient, clinician and others can do, and (4) agreeing actions. Further details of the intervention and training are reported elsewhere [6].

#### Control condition

In the control group, patients independently completed the same ratings on a tablet computer at the end of their routine meetings once a month for six months. There was no collaboration or further discussion with the clinician.

### Data collection

The primary outcome (subjective quality of life) and secondary outcomes, including sample size calculation, are reported in detail elsewhere [6]. This process evaluation reports on additional retrospective analyses.

#### Views of patients and clinicians

Qualitative data on the views and experiences of patients and clinicians in the experimental group were collected shortly after their final DIALOG+ session. Patients took part in focus groups based on a semi-structured interview schedule that was developed by the research team and piloted with service users. Clinicians also participated in focus groups or, where this was not practically possible, individual interviews. For clinicians, a semi-structured interview schedule was also developed by the research team based on the DIALOG+ procedure and piloted with trainers. Focus groups were preferred due to the benefits of interaction among members [17].

Upon enrolment to the main trial, all participants were asked for additional written informed consent to participate in the focus groups or interviews. All clinicians and 80% of patients in the experimental group agreed to participate (the remaining 20% refused). A convenience sample was then used whereby consenting participants were allocated to focus groups based on availability. All sessions were conducted at Community Mental Health Teams by one facilitator (EG, male) and one co-facilitator (SW or LK, both female), who made notes throughout, with no non-participants present. Researchers were Graduate Research Assistants trained in qualitative methods. Most participants had met the researchers previously during the recruitment and outcome assessments for the trial. All participants were made aware that the purpose was to share their opinion on their experience of DIALOG+ and researchers were looking for no right or wrong answers. Sessions lasted approximately 60–90 minutes, were audio-recorded and transcribed verbatim. No transcripts were returned to participants for comment and no repeat interviews took place. Data collection took place between July 2013 and May 2014, ending once the research team agreed that data saturation had been reached.

#### Content of DIALOG+ sessions

In the experimental group, the details of each DIALOG+ session were automatically recorded on the tablet computer. This included the agreed action items that were entered into the software by the clinician at the end of the 4-step approach. The data were synchronised to a server where they became available to the research team. This data reflects the content and outcome (i.e. the agreed solutions) of each DIALOG+ session over the 12 month study period. Equivalent data were not available in the control group.

#### SQOL assessment

The primary outcome in the trial was SQOL as measured on the MANSA [16] during meetings with blinded assessors at baseline, three, six, and 12 months. Patients in the experimental and control group rated their satisfaction with different life domains on 12 Likert scales from 1 (“couldn’t be worse”) to 7 (“couldn’t be better”). This includes a general satisfaction item (“how satisfied are you with your life as a whole today?”) and 11 life domains (e.g. mental health, job situation, accommodation). This process evaluation focused only on the 12 month SQOL assessment. This is because a consistent effect was found across all three time points in the trial [6] but only the 12 month data reflect longer term outcomes after all patients had finished receiving DIALOG+.

### Data analysis

#### View of patients and clinicians

An inductive thematic analysis was used to analyse the focus groups and individual interviews, whereby the identified themes were derived from the data at a semantic level (i.e. what participants explicitly said) [18]. The facilitator and co-facilitator familiarised themselves with the transcripts and then coded them line by line, identifying a list of initial codes and condensing these into themes. All themes were entered into database software for ongoing comparison and reference. The patient and clinician data were initially analysed independently using this inductive approach. Following this, all themes relating to the research question (i.e. the mechanisms of DIALOG+) were combined across both sets of data. The process was iterative whereby the themes were regularly revisited and discussed among independent analysts and the wider research team to minimise bias and improve validity. Participants did not provide feedback on the findings. The final themes were summarised using verbatim quotes, with a focus on those related to the mechanisms of DIALOG+.

#### Content analysis of DIALOG+ sessions

A content analysis [19] of the action item data was conducted to simultaneously explore: (1) the nature of the agreed actions (at the level of individual action items), and (2) the domains discussed (at the level of each DIALOG+ session). This approach was appropriate given the large number of action items, their concise nature, and the need to condense the data to summarise the content.

For the content analysis of the agreed actions, each individual action item was coded for two pre-determined themes: (i) the person responsible for completing the action; and within that (ii) the type of action. The codes within each overarching theme, however, were generated through an inductive approach. Initially, each action item was coded according to who was responsible for the action and the type of action using preliminary codes. The preliminary codes were then condensed further into common categories. The frequency of action items within each category was counted.

For the content analysis of the domains discussed, a pre-determined coding framework was used based on the 11 specific domains in the MANSA scale [16]. Each DIALOG+ session was coded one-by-one according to the domains that were explicitly discussed (e.g. mental health, physical health, job situation). The frequency of sessions addressing each domain was counted. This analysis was later combined with the quantitative analysis of the SQOL domains.

#### Quantitative analysis of SQOL domains

An exploratory analysis was conducted to identify which of the 12 MANSA items showed the largest effect of DIALOG+ compared to the control group, or if there was a consistent effect across all domains. This analysis included patients randomised to either the experimental or control group, with the outcome assessed at 12 months. Patients in the experimental group were excluded if they did not receive any DIALOG+ sessions. This allowed for better integration with the content analysis. Using the same methodology as the primary analysis [6], which was pre-specified in an analysis plan, this analysis was conducted at the level of the individual and based on available cases. Mixed-effects linear regressions were conducted for each MANSA item with a fixed effect for treatment group and the associated baseline value of the item, and a random effect for clinician to account for clustering. This analysis was combined with the content analysis to explore whether the effect was specific to the domains addressed or consistent across all domains. Given the exploratory nature, the focus was on the adjusted mean differences, although confidence intervals and significance levels are also reported.

## Results

**Fig 1:**
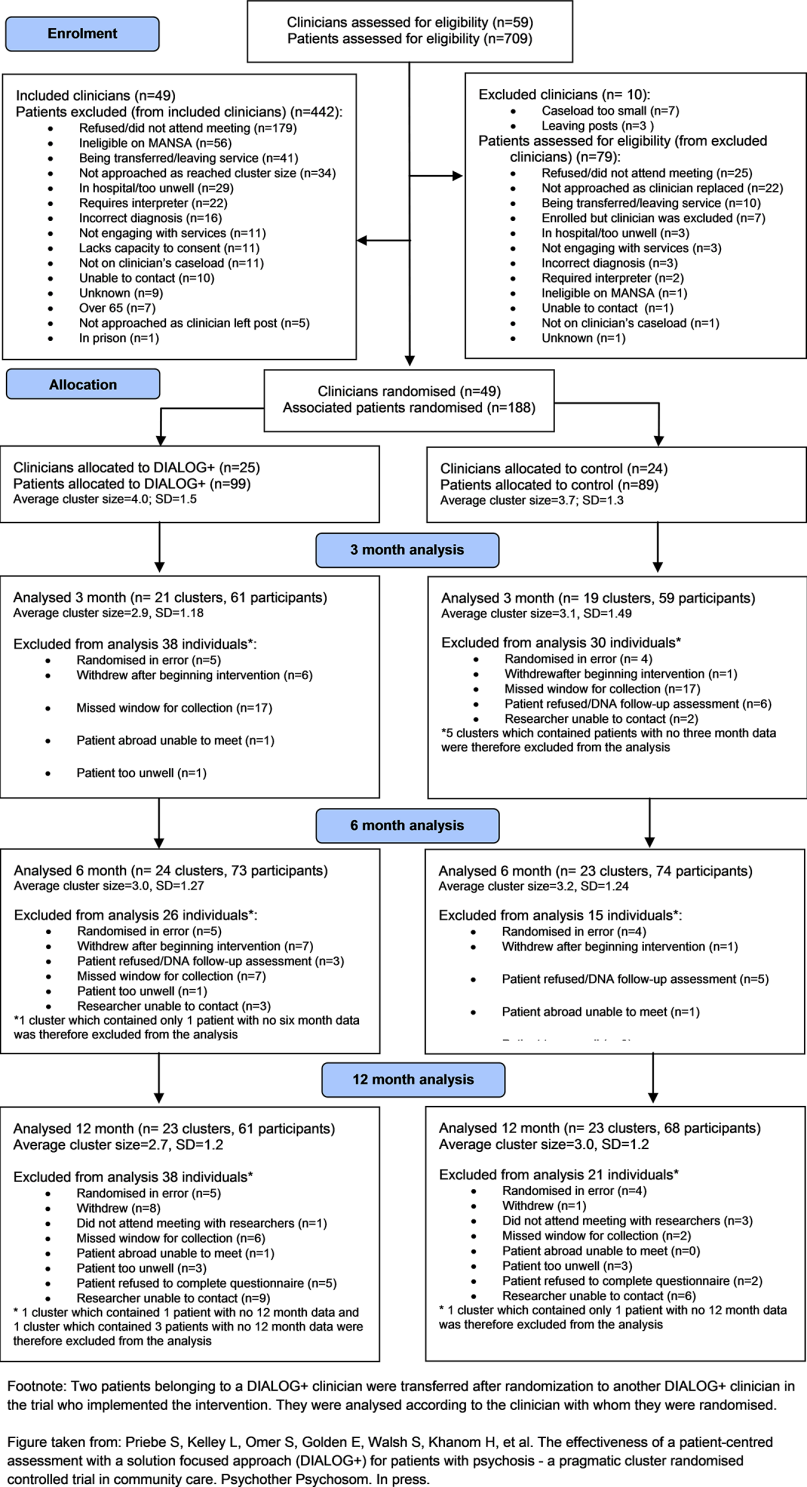
CONSORT flowchart.

Participant flow throughout the trial is shown in Fig 1. Detailed baseline characteristics for the trial are reported elsewhere [6]. In total, 49 clinicians (25 experimental, 24 control) were randomised with 179 patients (94 experimental; 85 control).

### Views of patients and clinicians

There were five patient focus groups (total of 19 patients) and four clinician focus groups plus five individual interviews (total of 19 clinicians).The participants were diverse across sessions with regards to gender, age and ethnicity. Four themes emerged regarding the mechanisms of DIALOG+: (1) a comprehensive structure; (2) self-reflection; (3) therapeutic self-expression; and (4) empowerment. These themes are described in more detail with quotes for illustration. Other responses that emerged related to the barriers to DIALOG+ (i.e. repetitiveness of the intervention, difficulty for clinicians to adhere, difficulty for patients to understand or cope with questions, and initial apprehension to the technology); but as they are not related to the research question they are not reported here in detail.

#### A comprehensive structure

This theme relates to the benefits of DIALOG+ as a comprehensive structure that forms the basis of routine meetings. Within this, many clinicians reported that the assessment of satisfaction provided a holistic structure that covers all aspects of the patient’s life, not just their mental health.

*It is actually very beneficial because you have your questionnaire that has been structured in such a way that you can cover almost everything that you need to cover with a client*, *from the psychosocial intervention to other things like their physical health*, *their mental health*, *their social life*, *their relationships…*(Clinician [C] 1, Focus Group [FG] 7)

In addition to the holistic assessment, some clinicians reported that the structure made the meetings more focused by setting an agenda based on important topics.

*[DIALOG+ was] a mechanism for formulating an agenda and*, *you know*, *letting them know that “we’ve got an hour together*, *let’s make the most out of what you need to talk about” and this enables us to do that … It just was a catalyst*, *it was a useful way to focus on relevant topics rather than irrelevant topics*. (C1, FG8)

Similarly, patients reported the benefits of DIALOG+ covering a wide range of topics.

*It was more structured and more focussed*. *… And it*, *and it… And*, *we discussed different issues*. *Like*, *it was more of a holistic sort of approach*? *Like*, *discussing all areas of mental health*. (Patient [P] 1, FG1)

*It was thorough and it seemed like we went over everything*. (P2, FG2)

Patients also reported that the structured approach facilitated the agreement of solutions by the end of the discussion, ensuring that change occurs.

*[DIALOG+] was more focussed*, *and*, *um*, *there was like actions at the end where*, *after we had discussed*, *the few topics that we chose…she would make actions where she would say*, *“What could be done about it*?*” So*, *she would make notes in*, *like*, *say “Contact so and so for this”… And I think that was better*, *because things got done*, *in that way… Issues got addressed… Constructive things were being done about certain issues*. *So I think more and more was being done*, *with [DIALOG+] in place*. (P1, FG1)

Some patients highlighted that the tablet computer itself was useful in facilitating this structure.

*It stopped your key worker forgetting everything… I did feel more in control when she was using the iPad I felt like we were using a structure*, *I felt more secure*. (P2, FG2)

#### Self-reflection

Many patients stated that DIALOG+, especially the assessment of satisfaction, encouraged self-reflection. They reported an increased awareness of their current situation and where changes were needed.

*Sometimes you get so caught up dealing with things on a daily basis that you don’t really check yourself*, *and when you’re asked these questions on a scale of 1–10 (sic) or whatever it kind of gives you more of an insight into how you are actually feeling*. (P3, FG3)

*It made me really stop and look at my life*, *and basically*, *like*, *the progress I need to make*, *or um*, *things that I need to stop doing … You make good use of the knowledge*, *that you need to improve yourself … Maybe prior to using [DIALOG+] it never actually crossed your mind*. *But the questions are so in your face that now you realise that*, *yeah*, *this is something that I need to work on*. (P4, FG4)

Additionally, patients found it useful to monitor changes over time and this helped them to identify improving areas, which also instilled hope.

*It helped me track my progress…*. *On a monthly basis…My lifestyle*, *my accommodation*, *my safety*, *medication*, *all those things*. (P3, FG4)

*Sometimes you get so caught up in life you’re just praying for the good days*, *so it’s nice to know that … you can reflect and say*, *“well*, *last month I was feeling shitty but this month I’m all right”*, *and it gives you a bit more hope for the future*, *so in that way it’s good*. (P3, FG3)

Clinicians also reported that the initial assessment helped patients to reflect on their situation and identify positive changes, which promoted hope and improved self-esteem.

*It gives them the opportunity to think*, *what makes it 3*, *how can it be 5*, *or 7*, *or even when it was 3 and now it’s 7*, *what has happened for it to go from 3 to 7 … It gives them thought to what has happened in the weeks or so since you’ve not met*. (C1, FG7)

*They would rate it*, *say*, *2*, *and then when you try to go*, *when you try to explore number 2*, *they say “oh*, *I should’ve been on 4” because they look at the negative aspect rather than the positive aspect of their lives … So it actually help them to think about the positive aspect rather than the negative aspects … You can actually have a look over the length of time where they are and they can actually see the positive aspect of what they’ve done … It shows them they’ve improved … It increases their self-esteem*. (C2, FG7)

#### Therapeutic self-expression

Some patients reported that DIALOG+ encouraged them to express themselves, which had a therapeutic impact. The process of exploring and expressing their thoughts and feelings on a wide range of topics helped improve their affect.

*Sometimes you’re… not feeling good inside and you’re holding things inside and… once you come in front of the computer*, *she would ask you questions and… that would… help you to express your feelings and things … I think it was the best way to… get yourself to express the feelings*, *held inside*. (P3, FG1)

*It’s like an offload*, *isn’t it*, *dust yourself off*, *you’re up to speed … It’s like you’ve been rebooted with [DIALOG+]*, *it’s like a different kind of therapeutic feeling… This cheers me up… I feel happy to do it*. (P2, FG5)

Clinicians also reported the benefits of DIALOG+ in encouraging patients to express themselves and this helped them to learn more about their patients.

*I thought it was really good … you got a lot of information*. *One client actually*, *I got so much more information*, *background information out of him because he really responded to this structure actually very well … A client who usually when you ask him “How are you feeling” usually answers “I’m fine” … he was giving away so much more about his past history*. (C2, FG6)

#### Empowerment

Many clinicians reported that DIALOG+ empowered their patients to become more involved in their treatment and decision-making.

*It was empowering because you are … shifting the care co-ordination responsibility and letting them make decisions … it was positive*. (C2, FG8)

*I found it the most empowering tool in the 10 years I have been qualified as a psychiatric nurse*. *By far*. *It helped me to empower my client … By the time we had got to the end*, *he had taken the reigns into his own hands … It definitely changed our therapeutic relationship… By the end really he was very very much in control of his own care*. (C4, FG6)

Clinicians reported that the solution-focused approach, especially, helped to shift the focus of the meetings towards the patient generating ideas and taking responsibility.

*Normally some of them will want you to do everything for them but with this solution focused therapy it did help them to see how to actually help themselves rather than being spoon-fed by the care coordinator … It did help them to understand how to actually come up with a solution rather than me saying “this is how to do it” … They come to you thinking “how can I do this” but … it’s like an eye-opener to some of them … They felt empowered to say that actually “I can do this by doing this or doing that*, *I can actually find a solution to my problem*.*”* (C3, FG7)

Some patients also reported a sense of empowerment, although this was less pronounced than the clinician interviews. This included an increased involvement in deciding which domains to talk about during the meetings.

*You would choose them from the list*, *yea*, *your care co-ordinator doesn’t choose anything for you*, *she gives you options*, *how do you feel*. (P2, FG5)

It also included increased reflection on what actions they can take to improve their situation.

*It can make you think about what you’re going to do for your life*, *obviously when you’re asked questions it can make you think about what you can do for yourself as well*. (P4, FG3)

### Content of action items

Of the 94 patients in the experimental group, 24 did not receive any DIALOG+ sessions and the data for 17 were unavailable due to technical problems in synchronising to the server. Thus, data on the DIALOG+ content over the 12 month study period were available for 193 unique sessions from 53 patients and 18 clinicians.

A total of 944 unique action items were identified. The mean number of action items per DIALOG+ session was 4.9. Table 1 summarises the person responsible and the nature of the action items. The patient was the responsible person for the majority of action items (65%). This was followed by clinicians (28%), family or friends (4%), and others (3%).There were a wide range of patient-led actions, with the most common related to healthier lifestyle (e.g. exercise, diet or substance misuse), engagement with treatment, or raising issues with healthcare professionals. The majority of clinician-led actions related to providing practical assistance with a problem (e.g. paying bills, completing forms), making a referral, or arranging a review of medication.

**Table 1:** The frequency of action items by responsible person and type of action.

Action item (responsible person/type of action)	N (%)
**Patient**	**617 (65.4)**
Healthier lifestyle	95 (10.1)
Engage with treatment	64 (6.8)
Request treatment or raise issue with clinicians	61 (6.5)
Attend service/appointment	60 (6.4)
Speak to or ask someone for help	58 (6.1)
Take part in recreational activity	55 (5.8)
Seek information	46 (4.9)
Manage symptoms/use coping techniques	41 (4.3)
Apply to or attend employment/education	39 (4.1)
Complete forms/paperwork	35 (3.7)
Domestic activity/shopping/budgeting	34 (3.6)
Consider options	20 (2.1)
Search for or move accommodation	9 (1.0)
**Clinicians**	**266 (28.2)**
Provide practical assistance	61 (6.5)
Make a referral	36 (3.8)
Arrange medication review	34 (3.6)
General support and encouragement	32 (3.4)
Change treatment approach	27 (2.9)
Liaise with other clinicians	26 (2.8)
Seek information	22 (2.3)
Provide supporting evidence	13 (1.4)
Provide reminders to patient	13 (1.4)
Speak to somebody else	2 (0.2)
**Family or friends**	**37 (3.9)**
Provide general support	24 (2.5)
Accompany to appointment/event	7 (0.7)
Provide practical assistance	6 (0.6)
**Housing staff or support worker**	**17 (1.8)**
Provide practical assistance	11 (1.2)
Change support/treatment approach	5 (0.5)
Accompany to appointments/events	1 (0.1)
**Patient and clinicians (joint responsibility)**	**5 (0.5)**
Domestic activity	3 (0.3)
Speak to employer	2 (0.2)
**Employer or education tutor**	**2 (0.2)**
Change to workload/tasks	1 (0.1)
Provide general support	1 (0.1)

### Content of DIALOG+ sessions and SQOL domains

The mean number of domains discussed per DIALOG+ session was 2.3 (SD = 1.1). This included a wide range of domains (see Table 2). The most commonly discussed domains were mental health, physical health, and accommodation.

**Table 2:** The frequency with which each domain was discussed in DIALOG+ sessions.

Domain	Number (%) of sessions
Mental health	120 (62.2)
Physical health	79 (40.9)
Accommodation	68 (35.2)
Job situation	56 (29.0)
Leisure activities	34 (17.6)
Friendships	32 (16.6)
Family	29 (15.0)
Financial situation	27 (14.0)
Personal safety	17 (8.8)
People that you live with	4 (2.1)

Of the 53 patients receiving a DIALOG+ session, 36 completed the MANSA at 12 months. Of the 85 patients in the control group, 68 completed the MANSA. The analysis of the individual MANSA items is summarised in Table 3. The treatment effect was greatest for accommodation (adjusted mean difference = 0.78), people with whom the person is living (= 0.73) and mental health (= 0.52). No significant effect was found on other domains, including general satisfaction. All three of these domains—living situation (accommodation and the people with whom the person is living) and mental health—were among those most commonly discussed in sessions (i.e. accommodation and mental health).

**Table 3:** Mixed effects linear regression for MANSA items at 12 months.

	Experimental		Control				
MANSA domain^1^	n	Mean(SD)	n	Mean (SD)	β	95% CI	p-value
Life as a whole	36	4.19 (1.37)	68	4.03 (1.40)	0.234	-0.289 to 0.756	0.377
Job situation	36	3.19 (1.51)	68	3.47 (1.71)	-0.184	-0.803 to 0.435	0.577
Financial situation	36	3.36 (1.38)	68	3.91 (1.61)	-0.301	-0.847 to 0.246	0.278
Friendships	36	4.39 (1.70)	68	4.09 (1.53)	0.303	-0.277 to 0.882	0.302
Leisure activities	36	4.14 (1.50)	68	3.87 (1.53)	0.071	-0.533 to 0.675	0.811
Accommodation	36	4.97 (1.58)	68	4.40 (1.70)	0.776	0.162 to 1.389	0.015
Personal safety	36	4.72 (1.43)	68	4.60 (1.28)	0.240	-0.314 to 0.795	0.383
People live with	36	5.36 (1.36)	68	4.84 (1.71)	0.725	0.017 to 1.433	0.045
Sex life	33	4.06 (1.71)	60	3.62 (1.53)	0.452	-0.176 to 1.081	0.156
Family	36	5.00 (1.45)	68	4.79 (1.55)	0.251	-0.332 to 0.834	0.384
Physical health	36	3.78 (1.57)	68	3.71 (1.56)	0.162	-0.493 to 0.817	0.620
Mental health	36	4.22 (1.25)	68	3.81 (1.71)	0.516	-0.059 to 1.090	0.078

Abbreviations.*β*: Beta coefficient (adjusted mean difference); *CI*: Confidence Interval; *MANSA*: Manchester Short Assessment of Quality of Life.

^1^ All MANSA items are analysed using mixed effects linear regression with treatment and baseline score fitted as fixed effects and clinician fitted as a random effect.

## Discussion

### Main findings

This process evaluation explored the possible mechanisms of DIALOG+. The treatment effect appears to be highest on particular areas (living situation and mental health), two of which were among those most commonly addressed during DIALOG+ sessions (i.e. accommodation, mental health, physical health). A wide range of action items were agreed during DIALOG+ sessions with patients taking responsibility for nearly two thirds of them (65%). Finally, the qualitative findings identified that DIALOG+: a) provided a comprehensive structure to routine meetings; b) helped patients to reflect on their current situation; c) encouraged therapeutic self-expression; and d) empowered patients to take control.

### Mechanisms of DIALOG+

Integration of these findings generates five hypothesised mechanisms through which DIALOG+ is effective.

#### Domain-specific change

The quantitative and content analyses suggest that the effect of DIALOG+ is, to an extent, domain-specific to two areas that are commonly addressed during sessions (accommodation and mental health). This is in contrast to a ‘general appraisal tendency’ whereby a consistent effect might be found across all subjective patient-reported outcomes, often more dependent on the patient’s overall mood than objective change [20–22]. DIALOG+ may instead bring about improvements to patients’ quality of life through addressing a specific concern and initiating positive change in that area. The thematic analysis of participants’ views supports this. Among the theme regarding a comprehensive structure, participants reported that DIALOG+ focused the discussion on the main issues and ensured constructive actions were agreed. Although physical health was also commonly addressed, and healthy lifestyle changes were the most frequent action items, there was no significant effect on this domain—perhaps due to the numerous barriers to improving such outcomes in this patient group [23].

#### A comprehensive, solution-focused structure

Previous attempts to improve routine meetings in community mental health care have had a limited effect, possibly because they fail to influence clinician and patient behaviour [3–5]. In contrast, DIALOG+ provides a model to directly guide the discussion that takes place. Simply incorporating such a model is important to maximise the therapeutic effect of any patient-clinician communication [24]. Both the thematic analysis and content analysis indicate that the DIALOG+ model is comprehensive, addressing a wide range of potential concerns. This includes practical issues as well as psychological ones, which will likely have a real impact on the patient’s life. Indeed, actions involving practical assistance (e.g. help paying bills or completing forms) were among the most commonly agreed during sessions. This holistic approach is important to address the complex range of health and social needs in this patient group [25].

The intervention also goes beyond an initial holistic assessment [26]. Participants reported that, in addition to setting an agenda for discussion, the structure encourages the identification of constructive solutions, ensuring that change occurs. This is reflected both in the moderate number of action items agreed (about five per session) and the apparent domain-specific effect. Although not central to the intervention, the computer technology may help to facilitate this comprehensive, solution-focused structure. This is in line with findings that a computer-assisted approach can maximise the effectiveness of therapeutic encounters [27, 28].

#### Self-reflection and therapeutic expression

Two unanticipated mechanisms that arose through the qualitative findings were self-reflection and therapeutic self-expression. Although perhaps not central to the domain-specific effect, these mechanisms might play a key role in improving general psychopathological symptoms (including depression and anxiety) as found in the main trial outcomes [6]. The initial assessment encouraged patients to reflect on their current situation and facilitated better insight into both the problematic and improving areas. Awareness of positive progress instilled hope which might have contributed to improved mood [29, 30]. The initial assessment also encouraged patients to express themselves which itself, as reported by patients, can have a therapeutic impact on affect [31].

#### Empowering patients

The content analysis found that patients receiving DIALOG+ took responsibility for the majority of agreed actions (65%). In comparison, previous findings showed patients are responsible for only a minority of actions (8%) in community mental health meetings [10]. This striking difference suggests DIALOG+ facilitates a shift toward the patient taking control. This is supported by the qualitative findings in which patients and clinicians commonly reported a change from clinician-led meetings towards empowering the patient.

The process of empowerment may be a key mechanism through which DIALOG+ initiates positive change. Empowerment itself is often identified by patients as important to recovery [32] and is associated with better quality of life and symptoms [33]. DIALOG+ appears to facilitate two key aspects of this process: participation in decisions and self-reliance [34]. Patients prefer shared-decision making [35] and a clinical orientation towards the patient taking more control can increase satisfaction [36]. Yet, patients often report a lack of involvement in treatment decisions [37]. The nature of DIALOG+ encourages patients to identify their own needs, goals and solutions and encourages them take more responsibility, both during treatment meetings and in the actions required to initiate change. This process of empowerment supports the benefits of DIALOG+ adopting a resource-oriented approach, whereby utilisation of the patient’s own strengths and resources is maximised [9].

### Strengths and limitations

This is the first process evaluation to explore the mechanisms of DIALOG+. The mixed-methods approach is a key strength, with triangulation of data sources (patient and clinician views, intervention content, patient-reported outcomes) and methodology (focus groups and interviews, content analysis, quantitative analysis) to explore both anticipated and unanticipated mechanisms. Adopting this approach enhances the interpretation of complex interventions [12, 15]. For example, on their own, the focus group and interview findings suggesting that DIALOG+ empowers patients to take control may only apply to a minority of patient-clinician pairs. However, the content analysis found that patients were responsible for the majority of actions, which strengthens this hypothesised mechanism.

Limitations of the trial are reported in detail elsewhere [6]. There are also limitations with the present process evaluation. The participants in the focus groups were a selection of all those who participated in the trial. Whilst we ensured that the focus group sample included participants with different characteristics, there may have been a selection bias. Data on the content of the meetings and action items in the control group were not collected. As a result, comparisons could only be made to the findings of previous research [10]. Although this did reveal large differences, only comparisons with a concurrent control could provide causal evidence for the role of DIALOG+ in changing the nature of routine community meetings. Additionally, this process evaluation focused on exploring possible mechanisms and did not investigate implementation or contextual factors that may influence the effectiveness of DIALOG+. The findings on the content of sessions and participant reports may also be prone to selection bias as they only include participants that used DIALOG+ over the intervention period. It is not clear from this study why some patients and clinicians failed to use DIALOG+ and if they would have used it in the same way. While data collection occurred in parallel with the trial, analyses were carried out post-hoc which may have been biased by knowledge of the trial outcomes.

### Implications and future research

This process evaluation has provided important insights into the possible mechanisms through which DIALOG+ impacts on patient outcomes. Specifically, it suggests that DIALOG+ effectively structures routine meetings making them comprehensive and solution-focused. It encourages self-reflection and therapeutic self-expression within the meetings. It also empowers patients to take control of their treatment, thus utilising their own strengths and resources. In doing so, DIALOG+ appears to bring about a domain-specific change to the areas addressed. Most of the patients in this trial had been in community treatment for many years. It needs to be tested as to whether similar processes are effective when DIALOG+ is applied to patients with shorter durations of psychosis–including patients with first episode psychosis–and to patient groups with other mental disorders.

Although the reported findings are exploratory, understanding how the intervention works can inform further developments and applications of DIALOG+. These mechanisms should be monitored in further research to establish and specify their mediating role. The identified mechanisms, such as empowerment, are not specific to the community treatment of patients with psychosis. This encourages the testing of DIALOG+ across other patient groups and settings. Future applications of DIALOG+, and indeed other therapeutic interventions, might consider these processes as important and strengthen them.

## Supporting Information

S1 COREQ Checklist COREQ checklist. (DOCX)

S1 GRAMMS Checklist GRAMMS checklist. (DOCX)

S1 Fig (DOCX)

S1 Protocol Trial protocol. (DOC)
