# Supplementary material for: Exploring the Mechanisms of a Patient-Centred Assessment with a Solution Focused Approach (DIALOG+) in the Community Treatment of Patients with Psychosis: A Process Evaluation within a Cluster-Randomised Controlled Trial
Source: PLoS One. 2016 Feb 9;11(2):e0148415. doi: 10.1371/journal.pone.0148415 (PMC4747516; doi:10.1371/journal.pone.0148415)
Supplement: S1 GRAMMS Checklist — (DOCX) [file pone.0148415.s002.docx]

**Good Reporting of A Mixed Methods Study (GRAMMS)**

| **Guideline** | **Section: page** |
| --- | --- |
| Describe the justification for using a mixed methods approach to the research question | Design: p5  Strengths and limitations: p23-24 |
| Describe the design in terms of the purpose, priority and sequence of methods | Design: p5 |
| Describe each method in terms of sampling, data collection and analysis | Data collection: p8-10  Data analysis: p10-12 |
| Describe where integration has occurred, how it has occurred and who has participated in it | Design: p5-6 |
| Describe any limitation of one method associated with the present of the other method | Strengths and limitations: p23-24 |
| Describe any insights gained from mixing or integrating methods | Discussion: p20-25 |

*O'Cathain A, Murphy E, Nicholl J. The quality of mixed methods studies in health services research. J Health Serv Res Policy. 2008;13(2):92-98.*
