# Supplementary material for: Exploring the Mechanisms of a Patient-Centred Assessment with a Solution Focused Approach (DIALOG+) in the Community Treatment of Patients with Psychosis: A Process Evaluation within a Cluster-Randomised Controlled Trial
Source: PLoS One. 2016 Feb 9;11(2):e0148415. doi: 10.1371/journal.pone.0148415 (PMC4747516; doi:10.1371/journal.pone.0148415)
Supplement: S1 Fig — (DOCX) [file pone.0148415.s003.docx]

**Screenshots of the DIALOG+ intervention**

1.
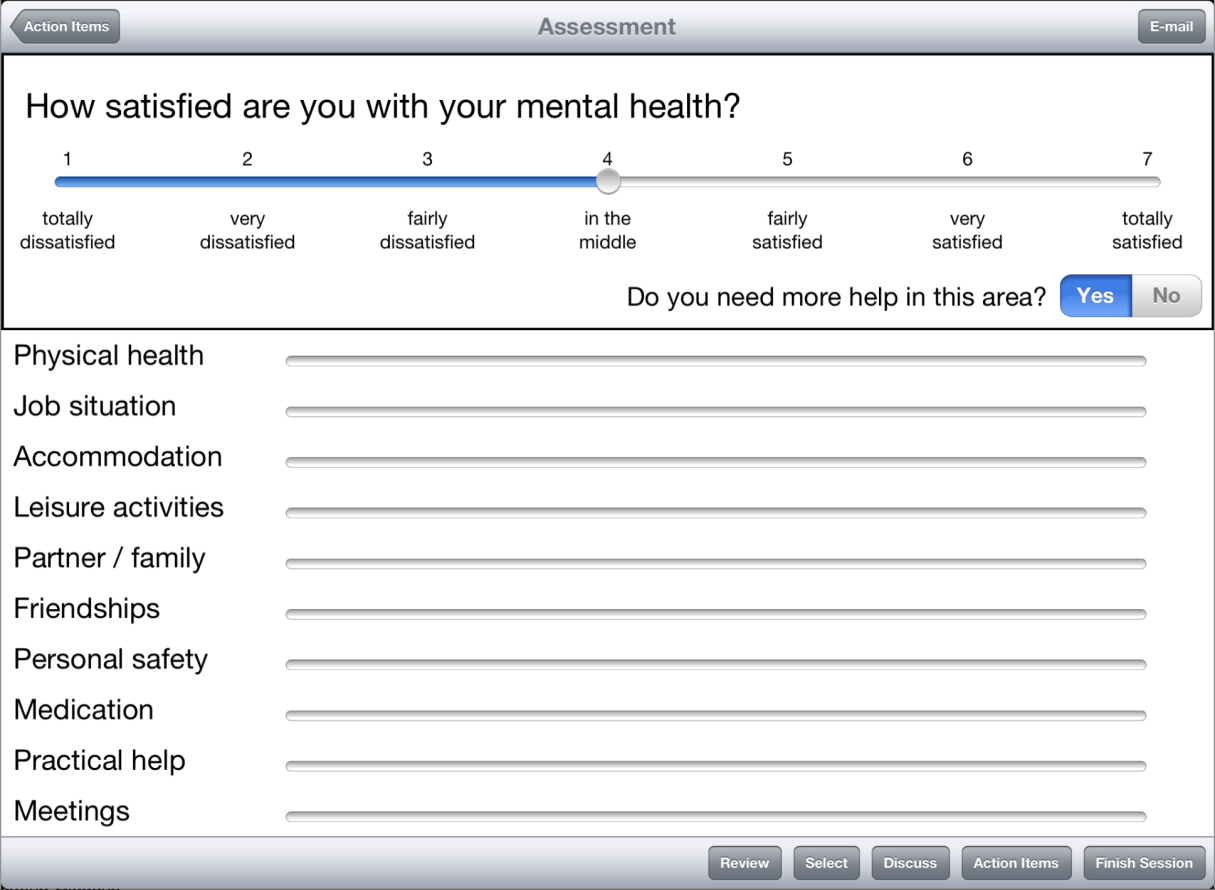
Screenshot of the initial structured assessment used in DIALOG+ as displayed on the tablet computer.
2.
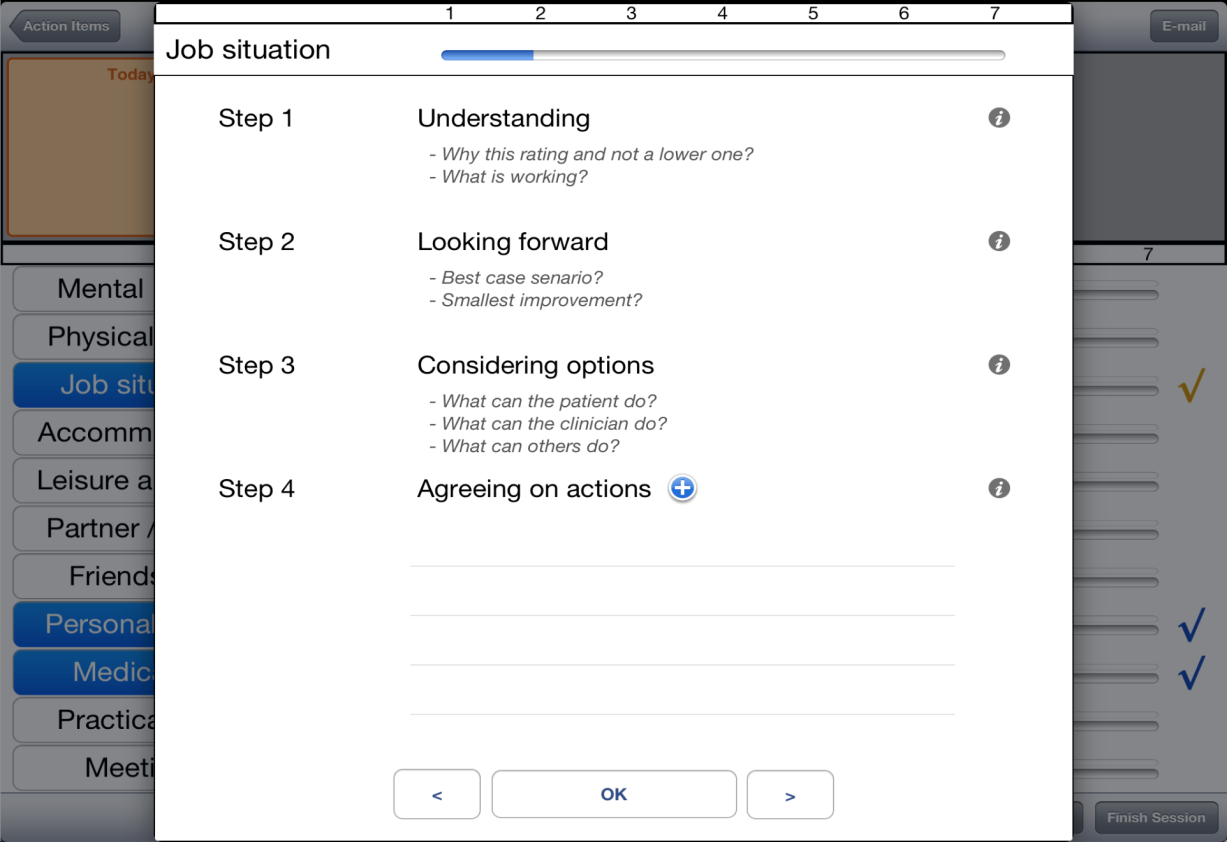
Screenshot of the 4-step approach as displayed on the tablet computer.
