# Supplementary material for: Exploring the Mechanisms of a Patient-Centred Assessment with a Solution Focused Approach (DIALOG+) in the Community Treatment of Patients with Psychosis: A Process Evaluation within a Cluster-Randomised Controlled Trial
Source: PLoS One. 2016 Feb 9;11(2):e0148415. doi: 10.1371/journal.pone.0148415 (PMC4747516; doi:10.1371/journal.pone.0148415)
Supplement: S1 Protocol — (DOC) [file pone.0148415.s004.doc]

Version: 2

Date of Finalisation: 31/05/2012

**TITLE OF THE PROTOCOL:**

The EPOS Trial: Effective Patient-Clinician Communication in Community Mental Health Care

**Short title:** The EPOS Trial

**Sponsor:** Queen Mary University of London

Representative of the Sponsor:

Gerry Leonard

Head of Research Resources

Joint Research Management Office

5 Walden Street

London

E1 2EF

Phone: 020 7882 7260

Email: sponsorsrep@bartshealth.nhs.uk

**REC reference:** TBD

**Chief Investigator Agreement Page**

The study as detailed within this research protocol (Version 2, dated 31/05/2012), or any subsequent amendments, will be conducted in accordance with the Research Governance Framework for Health & Social Care (2005), the World Medical Association Declaration of Helsinki (1996) and the current applicable regulatory requirements and any subsequent amendments of the appropriate regulations.

**Chief Investigator Name:** Stefan Priebe

**Chief Investigator Site:** East London NHS Foundation Trust

**Signature:**  **Date:** 31/05/2012

**
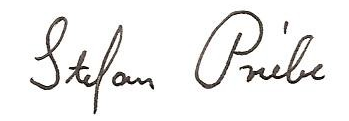
**

**STUDY SUMMARY/SYNPOSIS**

| **TITLE** | The EPOS Trial: Effective Patient-Clinician Communication in Community Mental Health Care |
| --- | --- |
| **SHORT TITLE** | The EPOS Trial |
| **Protocol Version Number and Date** | Version 2, Date 31/05/2012 |
| **Methodology** | Randomised control trial |
| **Study Duration** | 24 months |
| **Study Centre** | East London NHS Foundation Trust |
| **Objectives** | 1) To test whether regular use of the DIALOG+ intervention over a six month period improves patients' subjective quality of life.  2) To test whether DIALOG+ leads to improvements in recovery, objective social outcomes, social contacts, treatment satisfaction, the therapeutic relationship, needs, self-efficacy, psychopathological symptoms, and well being.  3) To test the cost-effectiveness of DIALOG+.  4) To investigate the extent to which clinicians adhere to the DIALOG+ manual, assess how it is implemented and explore the impact on the actual patient-clinician communication.  5) To gauge the experiences and opinions of clinicians and patients on the DIALOG+ intervention. |
| **Number of Patients** | 180. |
| **Main Inclusion Criteria** | Key workers  Professional qualification as a clinician (nurse, social worker, psychologist, occupational therapist, doctor);  More than 6 months experience of working in community mental health care;  Working as care coordinator.  Patients  Treatment in a community mental health care team in the NHS for at least one month;  No planned discharge for the next six months;  Clinical diagnosis of schizophrenia or a related disorder (F20-29);  Age between 18 and 65 years;  A mean score of lower than 5 on the MANSA;  Capacity to give informed consent. |
| **Method of Analysis** | Strict intention-to-treat or available case analysis |

**INDEX**

**Page**

**1. Introduction** **5**

1.1 Background 5

1.2 Importance 6

1.3 Rationale 6

**2. Study Objectives and Design** **7**

2.1 Study Objectives 7

2.2 Study Design 7

**3.** **Subject Selection** **9**

3.1 Subjects of interest 9

3.2 Number of subjects 9

3.3 Inclusion Criteria 9

3.4 Exclusion Criteria 10

**4.** **Study Procedure** **10**

4.1 Informed Consent Procedures 10

4.2 Data collection 10

4.3 Data management 11

4.4 Confidentiality 11

4.5 Compensation of Participants 11

4.6 Schedule of Treatment 11

4.7 Subject Withdrawal 11

4.8 End of Study Definition 11

4.9 Governance 11

**5.** **Analysis** **12**

**6.** **Publication Policy** **12**

**7.** **References** **12**

**1. Introduction**

**1.1 Background**

Approximately 1% of the population are affected by schizophrenia and related disorders with particularly high rates in urban areas (1). Such disorders result in significant distress for patients and carers and account for a substantial societal burden. They also generate high costs to the NHS through the need for ongoing intensive care and frequent hospitalisation, and to the society at large caused by loss of employment of patients and frequently also carers (2;3).

Patients with severe forms of schizophrenia are now regularly cared for in the community. As a result of major reforms of mental health care since the 1970s and substantial additional investments in the last 10 years, multi-disciplinary community mental health care teams have been set up throughout the UK and provide ongoing care. Every patient has a key worker (usually a nurse or social worker by background) who has regular meetings with the patient to assess their needs, engage them in treatment, discuss different treatment options and co-ordinate care. Yet, the interaction in these meetings is based more on common sense than on evidence based methods.

Whilst the evidence suggests that a more positive patient-clinician relationship is associated with more favourable outcomes (4-7), there is no evidence based intervention to achieve a better therapeutic relationship in community mental health care. Also, until recently there was no evidence based method to structure the communication between patient and clinician in a way that would eventually lead to more favourable clinical outcomes (8).

DIALOG is the first method to structure the patient-clinician interaction in community mental health care that has been shown to be associated with more favourable long term outcomes in a randomised controlled trial (9). In this computer-mediated intervention, patients are presented with fixed questions regarding their satisfaction with life and treatment and their needs for additional help. Subsequently, the ratings are displayed graphically and can be compared with ratings in previous meetings. In a cluster randomised controlled trial in six European countries, the DIALOG intervention was shown to be effective over a one year period. The intervention was associated with better subjective quality of life (SQOL), fewer unmet treatment needs and higher treatment satisfaction in patients with psychosis. The overall effect size was small. A stronger effect (medium effect size) was observed in the study site in the United Kingdom (East London) and in patients with more problematic baseline scores.

Following the trial, the National Institute for Health Research (NIHR) funded a programme to develop the intervention further both in terms of improving the technology and linking it with a simple psychological intervention informed by principles of Solution Focussed Therapy (SFT) and Cognitive Behavioural Therapy (CBT). The new intervention, combining the original intervention with a new 4-step approach to address problems raised by the patient, is called DIALOG+. The four steps consist of exploring the reasons for the dissatisfaction or wish for additional help and identifying positive coping skills; a forward looking consideration of best hopes and/or small changes; a discussion of all options for helpful actions by the patient, the clinician or other people; and an agreement of actions to be taken.

**1.2 Importance**

Patients diagnosed with schizophrenia or related disorders are the largest diagnostic group across all forms of secondary mental health services, and it can be estimated that more than 100,000 such patients are in care of community mental health care teams in England at any point of time (10). Established pharmacological and psychological treatments have only limited effect sizes in the long term treatment of schizophrenia, and are associated with substantial rates of non-adherence.

At present, patients regularly meet their key workers, but the patient-clinician interactions are not guided by evidenced based principles. A method to make these interactions more effective will not be a specialist programme for a small number of patients, but a generic method that can be utilised in routine care throughout the NHS. It does not require setting up new services or restructuring of organisations. It can be implemented at relatively low costs, particularly as it will not require extensive training of clinical staff, and can benefit tens of thousands of patients at the same time. Thus, even small health and social gains for individual patients will add up to substantial public health effects. This also applies to potential cost savings. The FOCUS study (in which patients’ outcomes were assessed by researchers and fed back to clinicians without structuring clinician-patient communication) suggested annual cost savings of regular outcome data feedback (which is also provided in DIALOG+) equivalent to £5172 per patient through reduced bed use (11). If replicated for only 20% of patients with schizophrenia and related disorders in community care in the NHS, the savings would exceed £100 million every year.

The procedure of DIALOG+ will also provide regular outcome data, i.e. patients’ ratings of satisfaction with life, treatment satisfaction and needs for further care. This data can be used to evaluate services on a local, regional and national level. So far, attempts to establish outcome assessment in routine community mental health care have largely failed, partly because it is difficult to motivate clinicians and patients to rate and enter outcome data on a regular basis. DIALOG+ provides a method to generate such data in way that is meaningful to clinicians and patients, and is likely to facilitate routine outcome assessment in secondary mental health services in the NHS.

**1.3 Rationale**

DIALOG+ differs considerably from the initial DIALOG intervention, warranting a new study. DIALOG+ involves a new version of the software (DIALOG 2.0) on a new platform (the iPad), further structures clinicians' behaviour according to the DIALOG+ manual, and involves training of clinicians. DIALOG+ aims to provide a way to deal with the specific concerns raised by the patient and, hence, equip the clinician as well as the patient with a method to explore and deal with problems.

Furthermore, we are seeking to conduct a more intensive intervention that will be delivered once a month over a six month period, rather than every two months over a one-year period, as in the original trial. We also wish to include a wider range of patients, as opposed to patients with persistent disorders who had been in care for an average of 15 years in the previous study. Finally, we wish to compare the intervention with a more defined control condition than treatment as usual, which will help to control for the effect of the implementation of an electronic device in the clinical setting and assessment of satisfaction, without providing feedback to the meeting, expanding on problematic domains or exploring needs for additional help. All these differences justify a new trial testing the effectiveness of DIALOG+.

**2. Study objectives and design**

**2.1 Study objectives**

1) To test whether the regular use of the DIALOG+ intervention over a six month period improves patients’ subjective quality of life.

2) To test whether DIALOG+ leads to patient improvements in recovery, objective social outcomes, social contacts, treatment satisfaction, the therapeutic relationship, needs, self-efficacy, psychopathological symptoms, and well being.

3) To test the cost-effectiveness of DIALOG+.

4) To investigate the extent to which clinicians adhere to the DIALOG+ manual, assess how it is implemented and explore the impact on the actual patient-clinician interaction.

5) To gauge the experiences and opinions of clinicians and patients on the DIALOG+ intervention.

**2.2 Study design**

An exploratory pragmatic cluster randomised controlled trial on the effectiveness and cost-effectiveness of DIALOG+. There is an additional qualitative component involving videotaping of patient-clinician dyads, and focus groups with patients and key workers.

Intervention to be tested

In the experimental group, DIALOG+ will be used as defined in the accompanying manual, and the DIALOG 2.0 software will run on an Apple iPad tablet. All clinicians in the experimental group will be trained in two sessions before the beginning of the intervention and receive a further two sessions for support and advice after about two months. Clinicians will be instructed to use DIALOG+ at least once per month over a six month period, although this is likely to vary due to the practical organisation of care. Clinicians and patients in the experimental group may decide to continue with DIALOG+ after the end of the six month intervention group. This will be documented and considered in the analysis of outcomes after the follow-up period.

Control condition

The control condition will include treatment as usual plus a defined intervention that also involves the use of an Apple iPad tablet and an assessment of the patient’s satisfaction. The patient will use the device to rate his/her satisfaction on the 11 domains of the DIALOG scale. The instruction will be to undertake such assessments after a meeting once per month and not to inform the clinician about the rating. This will help to control for the effect of the implementation of an electronic device in the clinical setting and assessment of satisfaction without providing feedback to the meeting, expanding on problematic domains or exploring needs for additional help.

Allocation to condition

Clinicians will be recruited from CMHTs in East London NHS Foundation Trust. Once clinicians have been recruited, patients meeting the eligibility criteria will be identified on their caseloads and randomly selected for participation in the study. Patients will then be asked by the clinicians for consent to be approached by a researcher. If patients consent, a researcher will contact them, do the baseline assessment and check all inclusion criteria. Due to ceiling/floor effects, patients who score very favourably on the primary outcome at baseline render a substantial improvement unlikely if not impossible. Therefore, patients who rate their mean subjective quality of life on the Manchester Short Assessment of Quality of Life (MANSA) (12) as 5 (satisfied) or higher will be excluded. Once five patients (per clinician) meeting the inclusion criteria have been recruited, the clinician – and correspondingly, his/her patients – will be randomly allocated to either the intervention or control group. Clustering of clinicians will prevent contamination effects in the study. Randomisation will be done by an independent statistician at the Pragmatic Clinical Trials Unit (PCTU) through e-mail.

Outcomes

Outcomes will be assessed at baseline and after 3, 6, and 12 months. We will attempt to keep the researchers assessing the outcomes masked about the allocation of the patients. If masking of the interviewer cannot be maintained, we will put researchers working at the Unit for Social and Community Psychiatry at the Newham Centre for Mental Health who are independent of the core research team)into contact with patients via telephone for conducting the assessment of the primary outcome and, as far as feasible, secondary outcomes. In such cases, patients will be asked not to discuss the treatment they received for the duration of the call.

The primary outcome will be subjective quality of life, measured on the MANSA (as in the original trial).

Secondary outcomes for the trial will consist of:

- Recovery as measured on the severity parts of each of the 24 items of the CHOICE scale (13) (satisfaction, i.e. the second part of each item, is already assessed on the MANSA)
- Objective Social Outcomes (SIX) (14), also assessed using the MANSA (sections 2 and 3), at 6 and 12 months only
- Social contacts in the last week in a structured interview
- Treatment Satisfaction on the Client Satisfaction Questionnaire (CSQ-8) (15)
- The Therapeutic Relationship on the Scale for Assessing Therapeutic Relationships in community mental health care (patient and clinician versions) (16)
- Needs on the Camberwell Assessment of Need Short Appraisal Schedule, patient-rated version (17)
- Self-Efficacy on the General Self-Efficacy Scale (18)
- Psychopathological Symptoms on the Brief Psychiatric Rating Scale (19)
- Costs of formal and informal care, assessed on the Client Service Receipt Inventory (20)
- Mental well-being on the Warwick-Edinburgh Mental Well-Being Scale (WEMWBS) (21).

Qualitative Assessments

For assessing the feasibility of the intervention, adherence to the manual and its effects in the patient-clinician meetings, each patient-clinician pair in the intervention group will have one of their meetings video-taped (or when unpractical audio-taped) and analysed. In the control group, we will do the same for 20% of the sample. Adherence to manual in the intervention group will be assessed against the manual for DIALOG+.

The software will automatically record what domains were explicitly discussed in DIALOG+ and what actions were agreed.

At the end of the intervention, separate focus groups will be conducted with patients and clinicians in the intervention group to obtain their experiences and suggestions for improvements.

**3. Subject selection**

**3.1** **Subjects of interest**

Patients

Patients in the East London NHS Foundation Trust, currently treated in a community mental health care team, with a clinical diagnosis of schizophrenia or a related disorder (F20-29).

Key workers

Key workers currently employed in the East London NHS Foundation Trust and working in community mental health teams.

**3.2 Number of subjects**

We will recruit 36 key workers and 180 patients, with 18 key workers and 90 patients in each of the conditions (five patients per key worker). Given the likely drop out of clinicians due to job changes or personal moves, we anticipate the loss of six key workers and 30 of their patients. With a patient drop out rate of less than 10% of the remaining 150 patients (as in the original trial it was less than 10% over a one year period) we will end with a sample of 136 patients. Assuming a practically negligible cluster effect (as in the original trial) the sample size will be sufficient to detect a medium effect size of 0.5 (Cohen’s D) with 80% power at the 5% significance level. A slightly higher effect of 0.57 will be detected with 90% power. The effect size of 0.5 would require that at the end of the intervention patients in the experimental group rate their satisfaction with at least 3 out of 12 life domains in the MANSA at least one point higher than patients in the control group. The sample size will also be sufficient to provide the required variation of experiences for addressing all other research questions.

Out of the caseload of all potentially eligible patients, we will randomly select 7 patients (allowing for 2 patients not fulfilling the inclusion criteria or not consenting to participate) per key worker. The remaining patients on the caseload of each key worker will be randomly ranked. They will be approached one-by-one if and when there is a need to recruit more patients for the given worker (e.g. in case more than 2 of the originally selected 7 do not consent to participate). Once all patients for one key worker have been recruited, the key worker will be randomly allocated to the intervention or control condition and receive either the training for DIALOG+ or the instructions for the control condition.

**3.3 Inclusion criteria**

To reflect the pragmatic nature of the trial, there will be wide inclusion criteria and very few exclusion criteria for both clinicians and patients. The inclusion criteria for patients will be wider than in the original DIALOG trial to test whether patients with more acute disorders, who have not been in care for many years and whose treatment in secondary services may not continue for another year, can also be recruited.

Patients

- Treatment in a community mental health care team in the NHS for at least one month;
- No planned discharge for the next six months;
- Clinical diagnosis of schizophrenia or a related disorder (F20-29);
- Age between 18 and 65 years; A mean score of lower than 5 on the MANSA;
- Capacity to give informed consent.

Key workers

- Professional qualification as a clinician (nurse, social worker, psychologist, occupational therapist, doctor);
- More than 6 months experience of working in community mental health care;
- Working as care coordinator.

**3.4 Exclusion criteria**

Patients

- Insufficient command of the English language for conducting meetings in English and filling in the assessment instruments of outcomes;
- A mean score of 5 or more on the MANSA;
- Learning difficulties.

Key workers

- None.

**4. Study procedures**

**4.1 Informed consent procedures**

Patients will be recruited from CMHTs in East London NHS Foundation Trust. Researchers will contact teams via e-mail and/or telephone to arrange a meeting to present the study to key workers, managers and consultant psychiatrists, and involving the leads at each site. One week after these meetings, a decision on participation will be sought. Written informed consent to participate will be obtained from individual key workers.

Clinicians, supported by research and administrative staff, will identify patients on their caseloads fulfilling the inclusion criteria, then ask suitable patients for consent to be approached by a researcher. If the answer is yes, a research assistant will provide an information sheet and arrange a meeting with the patient to discuss the project in full (at least two days later). At the meeting, the researcher will go through the information sheet, explain the study, obtain written informed consent, and establish all inclusion criteria. Once all patients (maximum of seven) from one clinician have consented, the clinician (and correspondingly, the clinician's patients) will be randomly allocated to either the intervention or control group. Randomisation will be done by an independent statistician at the Pragmatic Clinical Trials Unit (PCTU) through e-mail.

**4.2** **Data collection**

Data will be collected by the research assistants on the EPOS programme (NIHR Programme for Applied Research within which the trial will be conducted). We will seek adoption of the study on the Mental Health Research Network so that recruitment and baseline data collection can be supported by Clinical Scientific Officers of the MHRN. The researchers will keep ongoing contact with the clinicians and patients involved. There will be masking of researchers with respect to which group, intervention or control, the patients and clinicians belong to. For the assessment of the primary outcome at the end of the 6 month intervention period, they will be supported by other researchers in the Unit for Social and Community Psychiatry.

**4.3 Data management**

Data will be managed locally in the Unit for Social and Community Psychiatry complying with the standard operating procedures (SOPs) of the PCTU. The research assistants on the EPOS programme will be in charge of the data management on a day-to-day basis. Monitoring, quality assurance and archiving will also be conducted in accordance with the SOPs of the PCTU.

**4.4 Confidentiality**

All hard copies of data will be stored in a secure locked filing cabinet accessible only to the members of the project team. All electronic data will be stored on NHS or university computers, and patient identifiable information will be omitted upon data entry. Restricted access permission will be obtained for any documents containing patient identifiable information and these will be stored separately from the anonymised database. Only members of the project team will have access to this information. Video recordings and audio recordings will be stored in locked cabinets when not in use by the researchers. These recordings will only been seen/heard by members of the research team at the Academic Unit of Newham Centre for Mental Health and will not be played to anyone else. We will anonymise patients in the video recordings through blurring of faces.

**4.5 Compensation of participants**

Key workers

Key workers will participate in this research during their regular office hours and as part of their contracted work with the NHS Trust.

Patients

Patients will be paid £20 per clinical interview or focus group.

**4.6 Schedule of treatment**

In both the experimental and the control condition, patients will receive the given treatment condition, i.e. DIALOG+ or the independent rating of the DIALOG scale on an iPad, once per month for six consecutive months.

**4.7 Subject Withdrawal**

Participants will be informed through their consent forms that they are free to withdraw from the study at any time, without consequence, without having anything related to their services/employment affected. This fact will be emphasised by the researcher in all discussions explaining the study and prior to taking informed consent.

**4.8 End of Study Definition**

The end of the study will be when all project data for all patients and clinicians has been collected subsequent to the 6-month follow-up period, and data analysis is complete.

**4.9 Governance**

The trial will be subjected to all procedures of research governance in the participating NHS Trusts. The Trial Steering Committee is chaired by Prof. Liz Kuipers (King’s College London). Further members are Prof. Thomas Becker (University of Ulm, Germany), Prof. Tom Craig (King’s College London), Prof. Daniel Freeman (University of Oxford) and Prof. Lars Hansson (University of Lund, Sweden). A Data Monitoring and Ethics Committee will be established and the membership agreed with the Trial Steering Committee.

**5. Analysis**

Quantitative data will be analysed descriptively and by comparing outcomes in the two groups. Our primary analysis will be a strict intention-to-treat analysis or an available case analysis following intention-to-treat principles. Which analysis is used will depend on the number of missing values, the validity of any necessary assumptions (which may be particularly uncertain in this case) and the ease of multiple imputation (which will be used for the intention-to-treat analysis) given the analysis model. Currently, multiple imputation is, for example, much less straightforward if we opt for generalised estimating equations as our model of analysis. A decision about strict intention-to-treat or available case analysis will be made and written into a full analysis plan before any analysis is undertaken and before data are unblinded.

We intend to use generalised linear models as appropriate to the outcome, with fixed effects for the intervention and random effects for clinician (to adjust for the cluster effect of several patients treated by the same clinician). At end of treatment we expect missing data to be minimal. We will use sensitivity analyses to explore the impact of missing data.

Videotapes will be analysed according to specific criteria for adherence to manual. Audiotapes from focus groups will be analysed using thematic analysis.

**6. Publication Policy**

Data from this study will be anonymised and used in publications for peer reviewed scientific journals, conference presentations and publication on the Queen Mary University of London web site.

**7. References**

(1) Lloyd T, Kennedy N, Fearon P, Kirkbride J, Mallett R, Leff J, et al. Incidence of bipolar affective disorder in three UK cities: results from the AESOP study. British Journal of Psychiatry 2005;186:126-31.

(2) Gupta RD, Guest JF. Annual Cost of bipolar disorder to UK society. British Journal of Psychiatry 2002;180:227-33.

(3) Rodgers T. NICE recommends newer antipsychotic drugs as one of the first line options for schizophrenia. Nice Press Release. 2002.

(4) Klinkenberg WD, Calsyn RJ, Morse GA. The helping alliance in case management for homeless persons with severe mental illness. Community Mental Health Journal 1998;34:569-78.

(5) McCabe R, Priebe S. The therapeutic relationship in the treatment of severe mental illness: A review of methods and findings. International Journal of Social Psychiatry 2004;50:115-28.

(6) Priebe S, Gruyters T. The role of the helping alliance in psychiatric community care. A prospective study. The Journal of Nervous & Mental Disease 1993;181:552-7.

(7) Tattan T, Tarrier N. The expressed emotion of case managers of the seriously mentally ill: The influence of expressed emotion on clinical outcomes. Psychological Medicine 2000;30:195-204.

(8) Priebe S, McCabe R. The therapeutic relationship in psychiatric settings. Acta Psychiatrica Scandinavica 2006;429:69-72.

(9) Priebe S, McCabe R, Bullenkamp J. Structured patient-clinician communication and one-year outcome in community mental health care: A cluster randomised controlled trial. British Journal of Psychiatry 2007;191:420-6.

(10) Mental Health Strategies. Combined Service Mapping Framework. http://www.mhcombinedmap org/Reports aspx 2008.

(11) Slade M, McCrone P, Kuipers E, Leese M, Cahill S, Parabiaghi A, et al. Use of standardised outcome measures in adult mental health services. British Journal of Psychiatry 2006;189:330-6.

(12) Priebe, S., Huxley, P., Knight, S. & Evans, S. (1999). Application and results of the Manchester Short Assessment of Quality of Life (MANSA). *International* *Journal of Social Psychiatry*, 45:7-12.

(13) Greenwood, K.E., Sweeney, A., Williams, S., Garety, P., Kuipers, E., Scott, J. & Peters, E. (2010). Choice of outcome in CBT for psychosis (CHOICE): The development of a new service user–led outcome measure of CBT for psychosis. *Schizophrenia Bulletin*, 36:126–135.

(14) Priebe, S., Watzke, S., Hansson, L. & Burns, T. (2008). Objective social outcomes index (SIX): a method to summarise objective indicators of social outcomes in mental health care. *Acta Psychiatrica Scandinavica*, 118:57-63.

(15) Nguyen, T.D., Attkisson, C.C. & Stegner, B.L. (1983). Assessment of patient satisfaction: development and refinement of a service evaluation questionnaire. *Evaluation and Program Planning*, 6:299-313.

(16) McGuire-Snieckus, R., McCabe, R., Catty, J., Hansson, L. & Priebe, S. A new scale to assess the therapeutic relationship in community mental health care: STAR. *Psychological Medicine,* 37:85-95.

(17) Phelan, M., Slade, M., Thornicroft, G. & Parkman, S. (1996). The Camberwell Assessment of Need (CAN): comparison of assessments by staff and patients of the needs of the severely mentally ill. *Social Psychiatry and Psychiatric Epidemiology*, 31:109-113.

(18) Schwarzer, R. & Jerusalem, M. (1995). General Self-Efficacy scale. In J. Weinman, S. Wright, & M. Johnston, *Measures in health psychology: A user’s portfolio. Causal and control beliefs* (pp. 35-37). Windsor, UK: NFER-NELSON.

(19) Overall, J.E. & Gorham, D.R. (1962). The brief psychiatric rating scale. *Psychological Reports,* 10:799­-812.

(20) Beecham, J. & Knapp, M. (2001). Costing psychiatric interventions. In Thornicroft, G. (ed.), *Measuring Mental Health Needs*, 2nd edition (pp. 200-224). London, UK: Gaskell.

(21) Tennant, R., Hiller, L., Fishwick, R., Platt, S., Joseph, S., Weich, S., Parkinson, J., Secker, J., Stewart-Brown, S. (2007). The Warwick-Edinburgh Mental Well-being Scale (WEMWBS): development and UK validation. *Health and Quality of Life Outcomes*, 5:63.
